# The adenylate cyclase toxin RTX domain follows a series templated folding mechanism with implications for toxin activity

**DOI:** 10.1016/j.jbc.2023.105150

**Published:** 2023-08-09

**Authors:** Guojun Chen, Han Wang, Ladislav Bumba, Jiri Masin, Peter Sebo, Hongbin Li

**Affiliations:** 1 Department of Chemistry, University of British Columbia, Vancouver, British Columbia, Canada; 2 Institute of Microbiology of the Czech Academy of Sciences, v.v.i., Prague, Czech Republic

**Keywords:** protein folding, bacterial toxin, adenylate cyclase, single-molecule biophysics, optical tweezers

## Abstract

Folding of the Repeats-in-toxin (RTX) domain of the bacterial adenylate cyclase toxin-hemolysin (CyaA) is critical to its toxin activities and the virulence of the whooping cough agent *Bordetella pertussis*. The RTX domain (RD) contains five RTX blocks (RTX-i to RTX-v) and their folding is driven by the binding of calcium. However, the detailed molecular mechanism *via* which the folding signal transmits within the five RTX blocks remains unknown. By combining single molecule optical tweezers, protein engineering, and toxin activity assays, here we demonstrate that the folding of the RD follows a strict hierarchy, with the folding starting from its C-terminal block RTX-v and proceeding towards the N-terminal RTX-i block sequentially. Our results reveal a strict series, templated folding mechanism, where the folding signal is transmitted along the RD in a series fashion from its C terminus continuously to the N terminus. Due to the series nature of this folding signal transmission pathway, the folding of RD can be disrupted at any given RTX block, rendering the RTX blocks located N-terminally to the disruption site and the acylation region of CyaA unfolded and abolishing CyaA’s toxin activities. Our results reveal key mechanistic insights into the secretion and folding process of CyaA and may open up new potential avenues towards designing new therapeutics to abolish toxin activity of CyaA and combat *B. pertussis*.

Pertussis, also known as whooping cough, is a highly contagious respiratory illness (1, 2) caused by the Gram-negative coccobacillus *Bordetella pertussis* (1, 3). The pathogen secretes a pore-forming adenylate cyclase toxin-hemolysin (CyaA, ACT, or AC-Hly) as a key virulence factor involved in suppression of innate and adaptive immune defense mechanisms of the host (3, 4, 5). CyaA belongs to the so-called Repeats-in-ToXin (RTX) family of toxins, which include cytolytic toxins, metalloproteases, and lipases (6). RTX toxins all share a common feature—a C-terminal RTX domain (RD) that is composed of tandem nonapeptide repeats, rich in glycine and aspartate residues, having a consensus sequence of GGxGxDxxx, where x can be any amino acid (aa) residue. The RTX repeats fold into a Ca^2+^-loaded right-handed parallel β-helix structure known as the β-roll (6).

CyaA is a bifunctional toxin and exhibits both cell-invasive adenylyl cyclase enzyme (cytotoxic) and pore-forming (hemolytic) activities on a variety of cells (6, 7). It plays important roles in the early stages of respiratory tract colonization by *B. pertussis*. CyaA is a large multidomain protein containing 1706 aa residues (7, 8). According to their function, CyaA is divided into the following domains (Fig. 1*A*): an N-terminal adenylyl cyclase domain (ACD) that upon delivery into eukaryotic cells binds cytosolic calmodulin and converts cellular ATP to cAMP; a translocation region that helps to translocate ACD into target host cells; a hydrophobic region capable of inserting into host cell membranes to form oligomeric pores; an acylation region (AR) that contains two acylation sites critical for the function of CyaA; and a C-terminal RD followed by a noncleavable secretion signaling region (4, 5).

**Figure 1:**
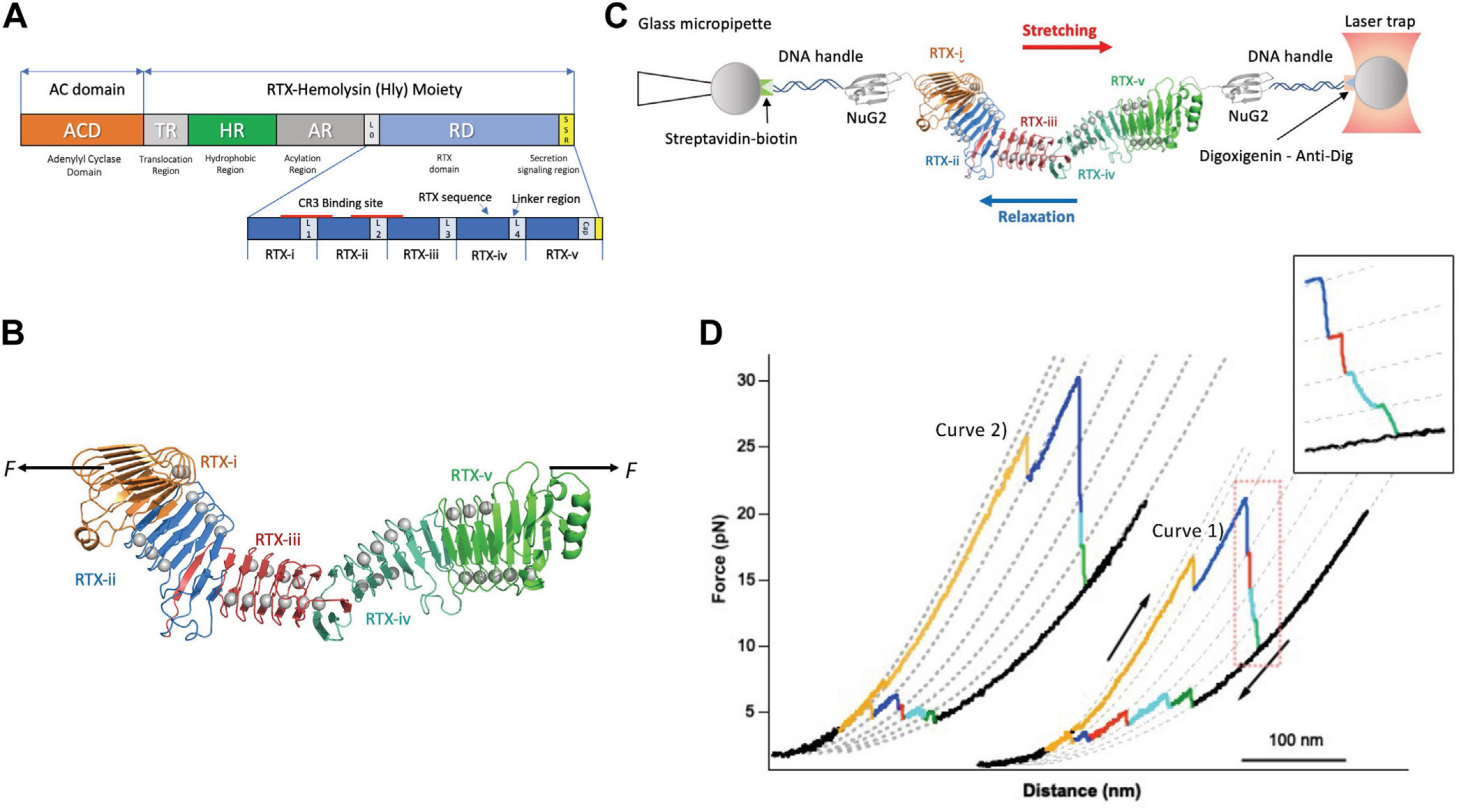
**Probing the unfolding and folding of RD using optical tweezer.***A*, scheme of the domain structure of CyaA. CyaA is a large multidomain protein that harbors different functional domains. RD is located at the C-terminal of CyaA and is comprised of five RTX blocks. Each block consists of RTX nonapeptide tandem repeat sequences followed by non-RTX linker sequence. The CR3-binding site binds CR3 integrin *via* two binding interfaces, which are between RTX-i and ii as well as between RTX-ii and iii. *B*, 3D structure of the RTX-i-v derived from the cryo-EM structure of RTX751 (PDB ID 7USL) (26). Ca^2+^ ions are shown as *gray spheres*. *C*, scheme of the OT experiment on NuG2-RTX-i-v-NuG2. NuG2-RTX-iii-v-NuG2 is coupled to two DNA handles (functionalized with biotin and digoxigenin, respectively) through thiol-maleimide reaction. The DNA-protein chimera is then captured by two polystyrene beads coated with streptavidin and anti-digoxigenin *via* specific ligand–receptor interactions. One bead is held by the glass micropipette and the other is trapped by the laser trap. By moving the laser trap, the investigated protein can be stretched and relaxed to induce unfolding and folding. *D*, representative F-D curves showing the unfolding-folding events of RTX-i-v. Individual (un)folding events are colored. *Dotted lines* are pseudo-WLC fits. When the second unfolding event (in *blue*) occurs at relatively lower forces, three additional unfolding events can be clearly resolved (curve 1 and inset). However, when the second unfolding event occurs at relatively higher forces, fewer unfolding events are resolved (curve 2). It is of note that the unfolding of the fingerprint domain NuG2 typically occurs at higher forces (Fig. S1). We often observed that RTX-i-v unfolds prior to NuG2, so that we can limit the stretching so that the unfolding of NuG2 does not occur, and F-D curves only display the unfolding-folding events of RTX-i-v. CR3, complement receptor 3; RD, RTX domain; RTX, Repeats-in-toxin.

CyaA is synthesized in the Ca^2+^-depleted bacterial cytosol as an intrinsically disordered protein that is secreted to the extracellular space by the type I secretion system (9, 10). Secretion of CyaA starts from its C terminus and RD is the first region to be secreted after the secretion signaling region. Outside the bacterial cell, the RD binds extracellular Ca^2+^ and folds into a Ca^2+^-loaded, functional assembly of five β-roll structures, which form the receptor-binding domain that mediates the binding of CyaA to the complement receptor 3 (CR3, or Mac-1, also known as α_M_β_2_ and CD11b/CD18) on host phagocytes (11). After CR3 binding, the CyaA inserts into the cytoplasmic membrane of host phagocytes and translocates the N-terminal enzymatic ACD across it directly into the host cell cytosol. In parallel, the membrane-inserted regions of CyaA can oligomerize into membrane pores that permeabilize the cells and enable potassium ion efflux (12, 13). The translocated ACD gets activated by binding cytosolic calmodulin and paralyzes cellular signaling of host cells by unregulated conversion of cytosolic ATP into the key cellular signaling molecule cAMP (6, 14, 15).

Previous studies showed that efficient secretion of CyaA to the extracellular milieu is critical to the virulence of *B. pertussis* and hinges upon the efficient secretion and subsequent folding of the RD of CyaA (16). The RD of CyaA (residue 1009–1681), which is the first region to be secreted, contains ∼40 more-or-less conserved nonapeptide repeats that are arranged into five RTX repeats blocks (RTX-i to RTX-v) (Fig. 1*A*). Despite RD’s critical importance, the understanding of the molecular mechanisms governing its secretion and folding process has just begun (16, 17), and the full mechanistic details remain to be elucidated.

It has been shown that the Ca^2+^-driven folding of RD accelerates CyaA’s secretion and mediates the formation of the CR3-binding site (11, 16, 18, 19, 20, 21, 22), which was shown to form at the interface between RTX-i and RTX-ii and between RTX-ii and RTX-iii (23, 24, 25, 26). In the presence of physiological Ca^2+^ concentrations (2 mM), the RD domain folds into a three-dimensional structure that consists of continuous β-roll structures interspersed with folded linker domains (Fig. 1*B*) (16, 26, 27, 28). It was shown that RTX-v folds vectorially in the direction from its C terminus to N terminus (16, 27), coinciding with RTX-v’s secretion direction. Based on the results on RTX-v, it was proposed that the whole RD may also follow this vectorial folding mechanism with the folding proceeding from its C toward N terminus (16, 27). However, this mechanism has not been tested or validated for the whole RD.

It is noteworthy that the C-terminal capping (C-cap) structure of RTX-v was found to be essential to the folding of RTX-v and the whole RD (16). Deleting residues 1636 to 1642 of C-cap (Δ1636–1642) not only rendered the whole RD and CyaA unfolded but also caused CyaA to lose its toxin activity and *B. pertussis* to lose its virulence in both *in vitro* and *in vivo* studies (16). Specifically, Δ1636–1642 disrupted CyaA’s ability to bind CR3 and disabled the delivery of the ACD into phagocytes, resulting in the loss of CyaA toxin activity and lethality of *B. pertussis* infection in mice. It is remarkable that the C-cap, which is distant from the CR3-binding site (located between RTX-i and RTX-iii, Fig. 1*A*) (11, 26), modulates CyaA’s binding to CR3 and its integrity determines the folding of the whole RD, suggesting that the C-cap of RTX-v regulates the folding and toxin activity of CyaA allosterically. However, the underlying molecular mechanism remains poorly understood.

Using RTX-iv-v as a model system, Motlova *et al.* (27) showed that RTX-iv alone is unfolded in the presence of Ca^2+^ but folds into a β-roll structure when linked to RTX-v. Based on this result, it was proposed that the folded RTX-v block may scaffold the Ca^2+^-driven cooperative stacking of nonapeptide repeats of its N-terminal RTX block (16, 27). Recent single molecule studies showed that indeed the folding of RTX-iv depends on the folding of RTX-v, both thermodynamically and kinetically, and the folding of RTX-iv is templated by RTX-v (29). However, the folding mechanism of the whole RD remains unknown. It is poorly understood how the folding signal is transmitted from the C to the N terminus of the RD and if the transmitting of the folding signal can be disrupted.

Due to the large size of RD (673 aa) and the seemingly similar thermodynamic stability of its RTX blocks (30), it is challenging to probe the folding mechanism of RD and investigate how the folding signal is transmitted along the RD using traditional biophysical tools. Indeed, CD spectroscopy, spectrofluorimetry, and NMR spectroscopy techniques are unable to discern the unfolding/folding events of each individual RTX block in RD. To overcome this challenge, here we combined single molecule optical tweezers (OT) with protein engineering and toxin activity assays to investigate how the folding signal is transmitted in the RD and in the AR that structurally represents an N-terminal extension capping the RD. We next examined the biological implications of disrupting this transmitting pathway of the folding signal.

Single molecule force spectroscopy (SMFS) techniques have evolved into a powerful tool to investigate the folding-unfolding dynamics of proteins at the single molecule level. SMFS allows one to stretch a protein of interest from its two specific residues to mechanically unfold and refold the protein and elucidate its unfolding-folding mechanism at the single molecule level. Among SMFS techniques, OT is of particular interest owing to its superb resolution in force and length (17, 31, 32, 33, 34). Using OT, we directly reveal that the folding of RD follows a strict hierarchy, with the folding starting from the C-terminal RTX-v and proceeding towards the N-terminus RTX blocks. Our results demonstrate a sequential, templated folding mechanism, *via* which the folding signal of RD is transmitted from its C terminus continuously to N terminus. In addition, the folding of AR is also likely governed by this templated folding mechanism. And the transmitting of the folding signal can be disrupted in RD and in turn affect the folding of RD and AR, as well as CyaA’s toxin activities. Our results have important biological implications and may open up new potential avenues towards designing new therapeutics to combat *B. pertussis*.

## Results

### The unfolding and folding of the whole RD occur in distinct individual steps

RD of CyaA contains ∼40 nonapeptide repeats that are arranged into five RTX repeats blocks (RTX-i to RTX-v) and folds into a continuous β-roll structure interspersed by linker regions (Fig. 1*B*). To investigate the folding mechanism of this large protein, we first used single molecule OT to stretch the whole RD domain from its N- and C-termini to probe its mechanical unfolding and refolding (Fig. 1*C*). Stretching RD resulted in the sawtooth-like force-distance (*F-D*) curves, where each individual sawtooth peak corresponds to one mechanical unfolding event. Up to five apparent unfolding force peaks were observed for RD in the *F-D* curves (Figs. 1*D* and S1). It is of note that the second unfolding event (colored in blue) occurred at high force (often >20 pN). If this unfolding event occurred at a relatively lower force (Curve 1 in Fig. 1*D*, and inset), three additional unfolding events could be clearly resolved following this unfolding event (Fig. 1*D*, inset). However, if this unfolding event occurred at a relatively higher force (curve 2 in Fig. 1*D*), fewer unfolding events were often observed. Evidently, if the unfolding force of the second unfolding event is high, some or all of the unfolding events 3 to 5 could be masked due to the limited resolution of OT, giving rise to fewer apparent unfolding peaks for RD. In this case, the second unfolding force peak involved the unfolding of multiple RTX blocks. The total contour length increment (*ΔLc*) upon unfolding of RD is ∼208 nm, which is comparable to the contour length of the fully unfolded RD (673 aa ∗ 0.36 nm/aa = 242 nm, where 0.36 nm/aa is the length of a single aa residue).

After unfolding, the unfolded polypeptide chain was then relaxed to allow RD to refold. The relaxation *F-D* curves showed sawtooth peaks at forces between 2 and 7 pN, with each peak corresponding to a refolding event. This result indicates that upon relaxation, the unfolded RD can refold against a force of a few pN. The refolding appears to occur largely in five steps, coinciding with the number of RTX blocks. Clearly, RD behaves as a multidomain protein and unfolds and refolds in multiple steps. However, due to the heterogeneity of the protein sequence, it is not possible to directly assign the unfolding or refolding events to specific RTX blocks in RD, and thus it is not possible to directly elucidate the transmitting mechanism of the folding signal in RD.

### RTX-iii unfolds and refolds in a two-state fashion and the folding of RTX-iii occurs after RTX-iv has folded

To investigate the folding mechanism of RD, it is imperative to distinguish and assign the folding and unfolding events to each individual RTX block, if the five RTX blocks indeed behave as individual blocks. To achieve this goal, we used a reductionist approach. Our previous OT experiments have established the mechanical unfolding and folding signatures of RTX-v and iv (17, 29). We revealed that RTX-v folds on its own, while the folding of RTX-iv is templated by the folded RTX-v, that is, RTX-iv folds only after RTX-v has folded. The unfolding and refolding of RTX-v and RTX-iv display distinct signatures: RTX-v shows a contour length increment *ΔLc* of 45 nm, while RTX-iv shows a *ΔLc* of 41 nm. The (un)folding of RTX-v can involve a stable intermediate state, while most of the unfolding and refolding events of RTX-iv involves two (un)folding intermediate states (Fig. S2). Moreover, in RTX-iv-v, RTX-v always folds prior to RTX-iv, and RTX-iv always unfolds prior to RTX-v (Fig. S3) (29). Based on these distinct signatures, we first sought to use RTX-iii-v to establish the unfolding/folding signatures of RTX-iii and examine if the templated folding mechanism also applies to RTX-iii.

**Figure 2:**
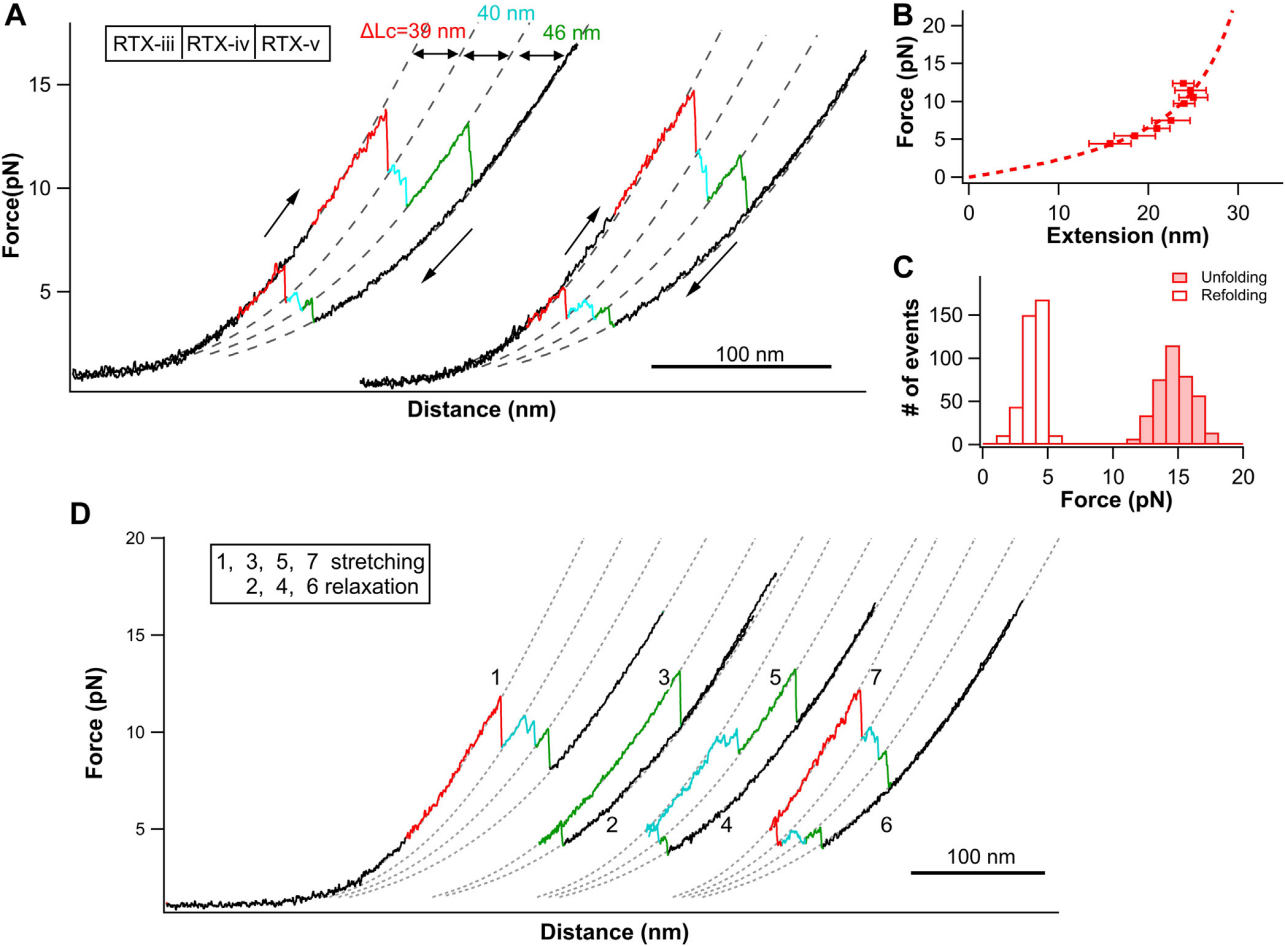
**Mechanical folding/unfolding of RTX-iii-v.***A*, representative *F-D* curves of RTX-iii-v in the presence of 10 mM Ca^2+^ at a pulling speed of 50 nm/s. Three distinct unfolding and refolding events are observed. The *red* unfolding events show a clear two-state unfolding behavior. Unfolding events of RTX-v can be readily identified by its characteristic ΔLc of 45 nm, while unfolding events of RTX-iii and iv show similar ΔLc. The unfolding events of RTX-iv can be identified by its characteristic multistate unfolding. The (un)folding events of RTX-iii, iv, and v are colored in *red*, *cyan*, and *green*, respectively. *B*, force-extension relationships of the (un)folding of RTX-iii. WLC fits to the data shows a ΔLc of 39.5 ± 0.6 nm. *C*, histograms of unfolding and refolding forces of RTX-iii at a pulling speed of 50 nm/s. The unfolding and refolding force is 14.1 ± 1.4 pN (average ± SD, n = 383) and 3.5 ± 1.0 pN, respectively. *D*, consecutive stretching-relaxation cycles allows for assigning the folding events to individual RTX blocks. F-D, force-distance; RTX, Repeats-in-toxin.

Figure 2*A* shows two pairs of representative *F-D* curves of RTX-iii-v in the presence of 10 mM Ca^2+^. Three groups of unfolding events were clearly observed (colored in red, cyan, and green, respectively). Fitting the force-length change relationship to the WLC model of polymer elasticity revealed that the three groups of unfolding events (colored in red, cyan, and green) showed a *ΔLc* of 39.0 ± 0.6 nm, 39.5 ± 0.6 nm, and 45.1 ± 0.7 nm, respectively (Fig. 2, *A* and *B*). From the distinct ΔLc of 45 nm, we can readily assign the green unfolding event to RTX-v. However, RTX-iii and iv showed similar *ΔLcs* (∼40 nm), making it difficult to discern these two unfolding events based on *ΔLc* alone.

It is of note that the first unfolding event (colored in red) occurred as a clear two-state unfolding event and no intermediate state was observed. In contrast, most of the cyan events involved intermediates. Evidently, the signatures of the cyan unfolding events matched those of RTX-iv (Figs. S2 and S3) (29). Therefore, based on the distinct unfolding behaviors, we can assign the cyan unfolding events to RTX-iv and consequently the red event to RTX-iii. Clearly, RTX-iii behaves as an apparent two-state unfolder, with an average unfolding force of 14.1 ± 1.4 pN (average ± standard deviation) at a pulling velocity of 50 nm/s (Fig. 2*C*). It is noteworthy that the unfolding of RTX-iii-v followed a strict order of RTX-iii, RTX-iv, and RTX-v.

Upon relaxation, the unfolded RTX-iii-v was observed to refold in three distinct events at ∼2 to 6 pN. Due to the low forces at which refolding occurred, the length change upon refolding was small, making it difficult to directly discern the refolding events. To address this challenge, we used a repeated stretching-relaxation strategy to assign the folding events. After RTX-iii-v was completely unfolded (curve 1, Fig. 2*D*), we relaxed the unfolded polypeptide chain until the first refolding event occurred (curve 2). This partially refolded RTX-iii-v was then stretched again to unfold the refolded block (curve 3). By measuring its *ΔLc* (∼45 nm), we can unambiguously assign the first refolding event to RTX-v. Similarly, completely unfolded RTX-iii-v was allowed to relax until two refolding events occurred (curve 4) and then stretched again to unfold these two refolded RTX blocks (curve 5). The cyan unfolding event displayed the distinct signatures of RTX-iv (multistate unfolding and *ΔLc* of ∼40 nm) and hence can be assigned to RTX-iv. It is worth noting that if only two RTX blocks were allowed to refold, RTX-iii was never observed to refold, as no unfolding event showing RTX-iii signatures was observed. Following the same strategy, the red refolding event can be assigned to RTX-iii (curve 6 and 7). By repeating this experiment for many cycles and also on different RTX-iii-v molecules, we observed that RTX-v is always the first to refold, followed by RTX-iv. And RTX-iii refolded only after RTX-iv had refolded. Evidently, the refolding of RTX-iii-v followed a strict hierarchical order of RTX-v → RTX-iv → RTX-iii, which started from the C terminus and proceeded towards the N terminus. This order is exactly the opposite of the unfolding hierarchy. The facts that RTX-iii does not fold independently and only folds after RTX-iv has folded suggested that the folding of RTX-iii is templated by the folded RTX-iv.

### RTX-ii shows higher mechanical stability but unfolds prior to RTX-iii-v and refolds after RTX-iii-v have refolded

**Figure 3:**
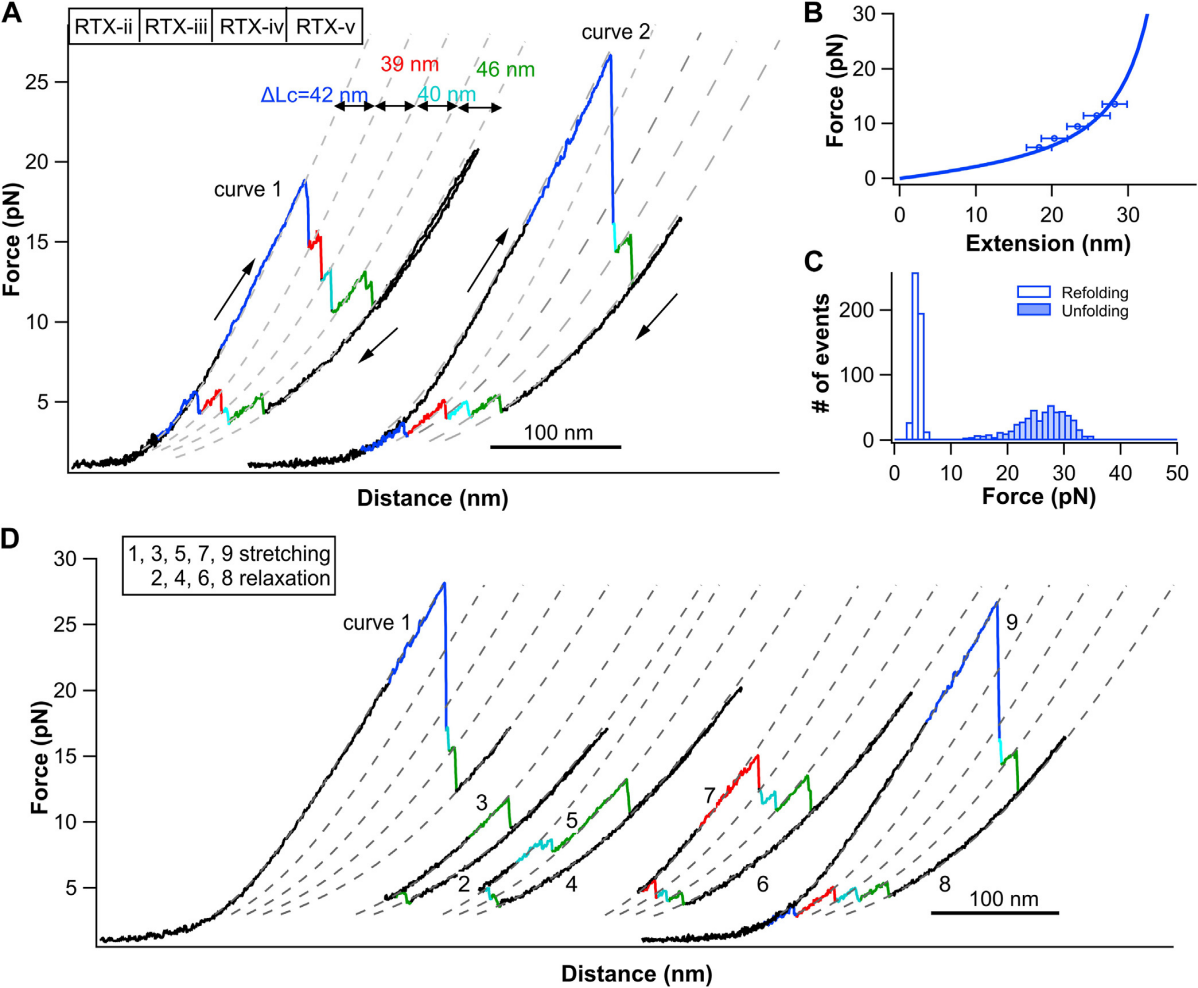
**Mechanical unfolding/folding signatures of RTX-ii.***A*, representative *F-D* curves of RTX-ii-v. Individual (un)folding events are colored. *B*, force-extension relationship of the (un)folding of RTX-ii. WLC model fitting yields a ΔLc of 41.6 ± 0.6 nm. *C*, histograms of unfolding and folding forces of RTX-ii at a pulling speed of 50 nm/s. The average unfolding force and refolding force are 26.3 ± 4.3 pN and 3.4 ± 1.0 pN, respectively. *D*, repeated stretching-relaxation curve of RTX-ii-v allows for discerning the unfolding and folding order of the RTX blocks. F-D, force-distance; RTX, Repeats-in-toxin.

Using the same strategy, we then investigated the unfolding-refolding of RTX-ii-v. Different from RTX-iii-v, the *F-D* curves of RTX-ii-v are characterized by a large unfolding force peak often occurring at >20 pN, which always occurred as the first unfolding event (Fig. 3*A*). Given that this high unfolding event is absent from RTX-iii-v but appears in RTX-ii-v, this first unfolding event can be readily assigned to the unfolding of RTX-ii. It is of note that if the unfolding event of RTX-ii occurred at relatively lower forces, three subsequent unfolding events (colored in red, cyan, and green) could be clearly observed (curve 1, Fig. 3*A*). However, if the unfolding force of RTX-ii was relatively high (curve 2, Fig. 3*A*), fewer unfolding events were observed. This behavior is similar to that observed for RTX-i-v. Evidently, the unfolding events of RTX-iii and iv were not fully resolved in curve 2. This is due to the fact that after the unfolding of RTX-ii, the residual force acting on the polypeptide chain was so high that RTX-iii and RTX-iv unfolded immediately after RTX-ii unfolded. The unfolding of RTX-ii shows a *ΔLc* of 41.6 ± 0.6 nm and an average unfolding force of 26.3 ± 4.3 pN at a pulling speed of 50 nm/s.

During the relaxation, the unfolded RTX-ii-v was observed to refold in four clear refolding steps (Fig. 3*A*). Using the same repeated stretching-relaxation strategy (Fig. 3*D*), we discerned and assigned the refolding events to individual RTX block. As shown in curves 1 to 7, when only three RTX blocks were allowed to refold, the high unfolding force event at >20 pN did not appear, suggesting that RTX-ii is the last RTX block to refold. By comparing the unfolding-refolding features of the first three refolded RTX blocks with those of RTX-iii-v (Fig. 2), we can readily assign the first three refolding events to the refolding of RTX-v (green), iv (cyan), and iii (red), respectively. As soon as the fourth RTX block was allowed to refold, the large unfolding force peak appeared in the subsequent stretching curve, and often the unfolding events of RTX-iii-iv were masked (curve 8 and 9, Fig. 3*D*). These results allowed us to assign the fourth refolding event (blue) to the refolding of RTX-ii.

These results again revealed that the unfolding of RTX-ii-v starts from the N terminus and proceeds in the direction of RTX-ii to RTX-v, while the refolding proceeds in the reverse direction from RTX-v to RTX-ii and occurred one by one sequentially. It is important to highlight that the unfolding sequence of RTX-ii-v did not follow the typical mechanical unfolding hierarchy, that is, the mechanically weakest domain unfolds first and strongest domain unfolds last in a tandem modular protein (35). Instead, RTX-ii, which has the highest unfolding force, always unfolds first. This abnormal, reversed unfolding hierarchy was observed previously in individual folded proteins (36, 37) as well as domain insertion proteins in which one protein is inserted in the middle of another protein (38). This abnormal unfolding hierarchy observed in RTX-ii-v suggested that RTX-ii-v cannot be considered as a tandem modular protein, in which individual domains behave largely independent. Instead, strong interactions must exist between individual RTX blocks. Although RTX-ii exhibits highest unfolding force, RTX-iii-v must be stabilized by folded RTX-ii so that RTX-iii-v is mechanically more stable than RTX-ii in the fully folded RTX-ii-v. Similar behaviors were observed in RTX-iv-v and the underlying stabilizing mechanism was validated experimentally (29). These results clearly revealed the importance of inter-RTX block interaction on the biophysical (and potentially functional) properties of RTX blocks.

### The folding of RTX-i-v is a vectorial folding process proceeding from the C terminus to the N terminus

**Figure 4:**
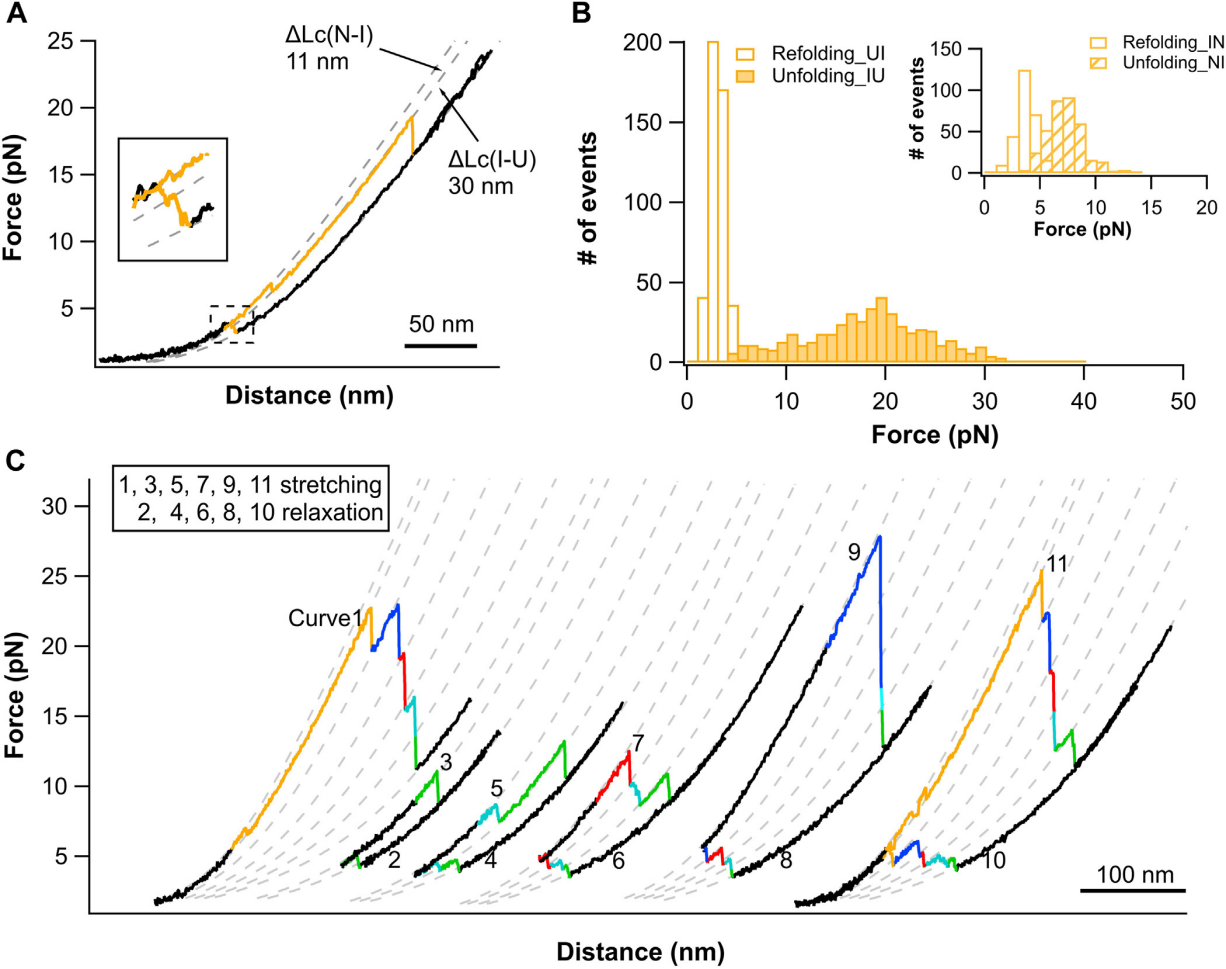
**RTX-i is the first RTX block to unfold but the last to refold.***A*, F-D curves showing the unfolding-refolding event of RTX-i. RTX-i unfolds and refolds *via* an intermediate state. *Dotted lines* are pseudo WLC fits that help identify the intermediate. *B*, unfolding and refolding force histogram of RTX-i. *C*, repeated stretching-relaxation curves of RTX-i-v shows that the unfolding of RTX-i-v occurs in the order of RTX-i to RTX-v, while the refolding occurs in the reverse order from RTX-v to i. F-D, force-distance; RTX, Repeats-in-toxin.

Having established the folding signatures of RTX-ii-v, we then discerned and assigned the (un)folding event of RTX-i based on the *F-D* curves of RTX-i-v (Fig. 1*C*). By comparing the *F-D* curves of RTX-i-v and of RTX-ii-v, it became evident that the unfolding event colored in orange can be readily assigned to RTX-i. The unfolding of RTX-i occurs in a clear three-state manner involving a stable unfolding intermediate state (Fig. 4*A*): the initial unfolding occurred at ∼4 pN with a *ΔLc* of 11.5 ± 0.3 nm, and the unfolding of the intermediate state occurred at ∼18 pN with a *ΔLc* of 29.7 ± 0.2 nm (Fig. 4*B*). By unfolding and refolding of only RTX-i, we observed that the folding of RTX-i appeared to be the reverse of the unfolding, involving a folding intermediate state (Fig. 4*A*). Repeated stretching-relaxation experiments (Fig. 4*C*) clearly revealed that RTX-i is the last one to refold and the first one to unfold in RTX-i-v.

Collectively, these results showed that the refolding of RTX-i-v in the presence of Ca^2+^ follows a strict vectorial folding pathway, and the folding starts from the C-terminal RTX-v and proceeds to the N-terminal RTX-i in a strict hierarchical, stepwise fashion. The folding of each individual RTX block occurs only after its C-terminal RTX block has folded, suggesting that its folding is templated by its C-terminal folded block. In turn, its folding can then template the folding of its N-terminal neighboring block. The folding signal is thus transmitted from the C- to the N-terminus in a series fashion. It is important to note that the folding signal does not jump or skip any RTX block in the native full-length RTX-i-v or N-terminally truncated RTX fragments (such as RTX-ii-v). Although previous studies showed that truncated RD variants, in which some nonapeptide repeats are deleted (for example, residues 1295–1561 are deleted in RD) (39), and hybrid fusion RTX variants, such as the construct in which the C-terminal RTX-v sequence is fused to RTX-i-iii (28), are folded, we never observed any folding trajectory in which one of the RTX blocks did not fold while its N-terminal neighboring RTX block folded. For example, we never observed any folding trajectory that contains a refolded RTX-v and RTX-i-iii but an unfolded RTX-iv. These results suggest that the templating effect we observed here is between neighboring RTX blocks in RD and long-range templating effect between non-neighboring RTX blocks, although possible, does not occur in the native RD, likely due to the high entropy penalty in closing an unfolded RTX block between two refolded non-neighboring RTX blocks (40, 41).

### The transmitting of the folding signal in RD can be disrupted, leading to the abolishment of CyaA toxin activity

The vectorial folding mechanism of the RD indicates that the folding signal is transmitted continuously along the polypeptide chain of the RD from its C-terminus to N-terminus, that is, the transmitting of the folding signal is a series process. This would suggest that disrupting the folding of any given RTX block could lead to the disruption of the folding of its N-terminal RTX blocks and thus block the transmitting of the folding signal in RD. This mechanism has important biological implications.

The binding of CyaA to the CR3 integrin is critical for CyaA toxin activity. Previously, the CR3-binding site was mapped to the interface between RTX-ii and iii (11). A recent cryo-EM structural study indicated that the CR3 integrin forms an extensive interface with RD that involves the linker regions between RTX-i and ii and between RTX-ii and iii (26). These new results suggested that the CR3-binding site is effectively encompassing RTX-i to iii. Hence, disrupting the folding of any RTX block should interfere with the correct assembly of the CR3-binding site, as disrupting the folding of any RTX block among RTX-ii to v, which will also disrupt the folding of its N-terminal blocks, should completely disrupt the fold of the CR3-binding site, while disrupting the folding of RTX-i should disrupt the binding interface mediated by RTX-i and ii. The allosteric effect observed in the C-cap of RTX-v supports this reasoning, as the C-cap deletion mutant Δ1636–2642 disrupts the CR3-binding site of CyaA and abolishes CyaA toxin activity (16).

To rigorously test this reasoning/hypothesis, we sought to engineer RTX variants that contained folding-impaired RTX blocks and examined their structural and functional consequences on CyaA. Although the detailed molecular mechanism remains unknown for the templating effect between neighboring RTX blocks, the interface between the two RTX blocks likely plays important roles. Based on the 3D structure of RTX-iv-v (PDB code: 6SUS) (27), we reasoned that if we inserted residues between RTX-iv and v, the inserted residues may cause a shift of the first β-strand in the β-roll structure of RTX-iv or interfere with the formation of the interface and consequently interfere with the proper folding of RTX-iv. Through trial and error, we discovered that an insertion of Arg-Ser (RS) in the linker between RTX-iv and v fulfilled this goal.

**Figure 5:**
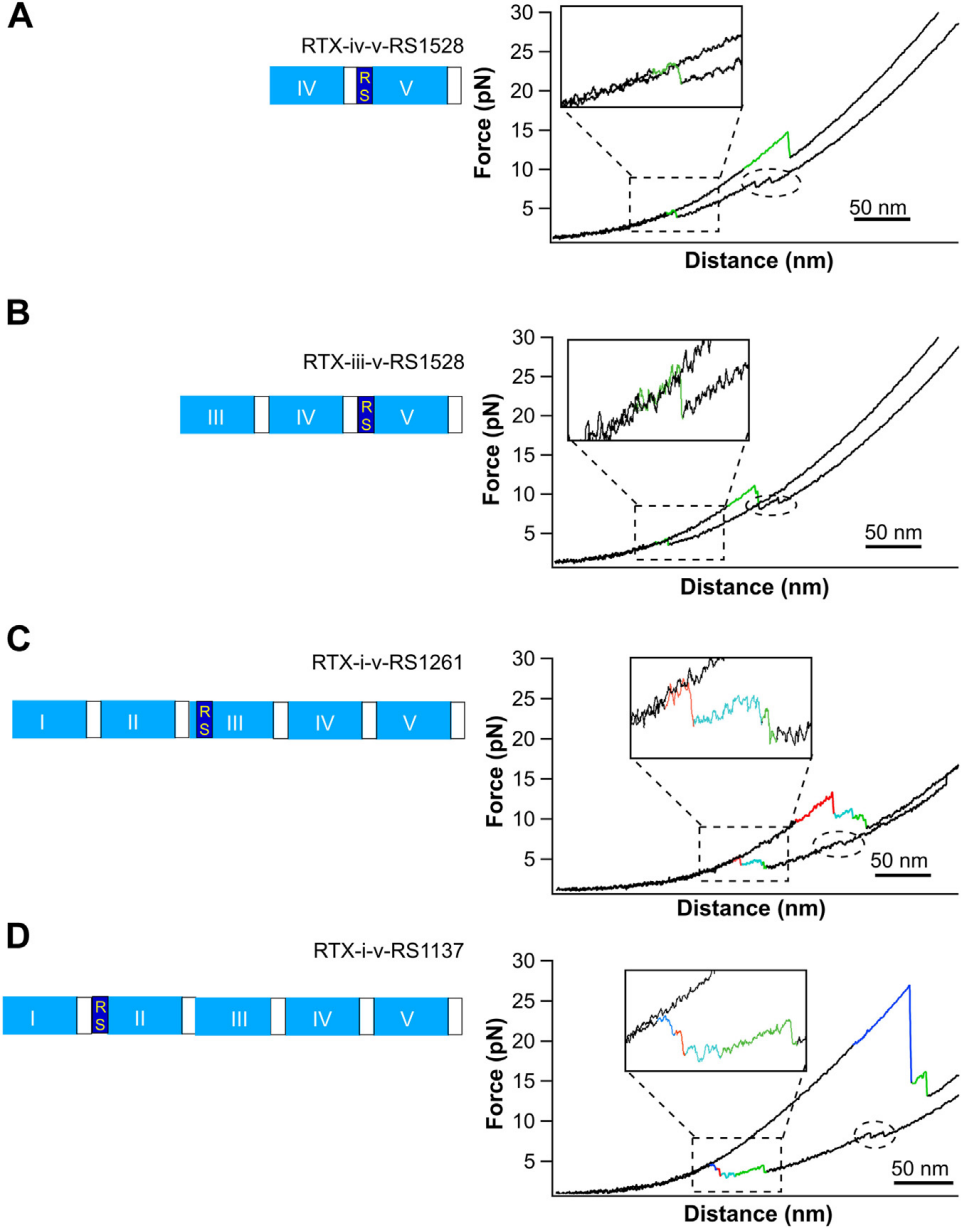
**The transmission of the folding signal can be blocked by the RS insertion**. *A*-*D*, the schematic of the domain structure (*left*) and the representative F-D curves (*right*) of different RS insertion RTX variants (A: RTX-iv-v-RS1528; B: RTX-iii-v-RS1528; C: RTX-i-v-RS1261; D: RTX-i-v-RS1137). In the schematics, RTX blocks are colored in *cyan*, and RS insertions are colored in *blue*. In F-D curves, unfolding and folding events are colored according to their identities, that is, RTX-v in *green*, RTX-iv in *cyan*, RTX-iii in *red*, and RTX-ii in *blue*. Zoomed view of the folding events of RTX blocks are shown in insets. Folding events of the fingerprint domain NuG2 are indicated by *circles*, while unfolding events of NuG2 occurred at higher forces (>30 pN). F-D, force-distance; RTX, Repeats-in-toxin.

By inserting RS between RTX-iv and v at position 1528, we engineered RTX-iv-v-RS1528. Figure 5*A* shows a representative *F-D* curve of the construct NuG2-RTX-iv-v-RS1528-NuG2 in the presence of 10 mM Ca^2+^, in which only the unfolding and refolding events of RTX-v and the fingerprint domain NuG2 were observed. The lack of unfolding or refolding events of RTX-iv indicated that RTX-iv was unfolded in 10 mM Ca^2+^, suggesting that the RS insertion disrupted the transmission of the folding signal through the linker to RTX-iv. To examine if the folding-impaired RTX-iv can disrupt the folding of its N-terminal RTX-iii, we then engineered NuG2-RTX-iii-v-RS1528-NuG2. As expected, the *F-D* curves of NuG2-RTX-iii-v-RS1528-NuG2 only showed the unfolding and refolding events of RTX-v and NuG2, while the (un)folding events of neither RTX-iv or iii were observed (Fig. 5*B*). This result clearly indicated that both RTX-iii and iv were unfolded in RTX-iii-v-RS1528, demonstrating that the folding-impaired RTX-iv disrupted the transmitting of the folding signal to RTX-iii and prevented RTX-iii from folding.

**Figure 6:**
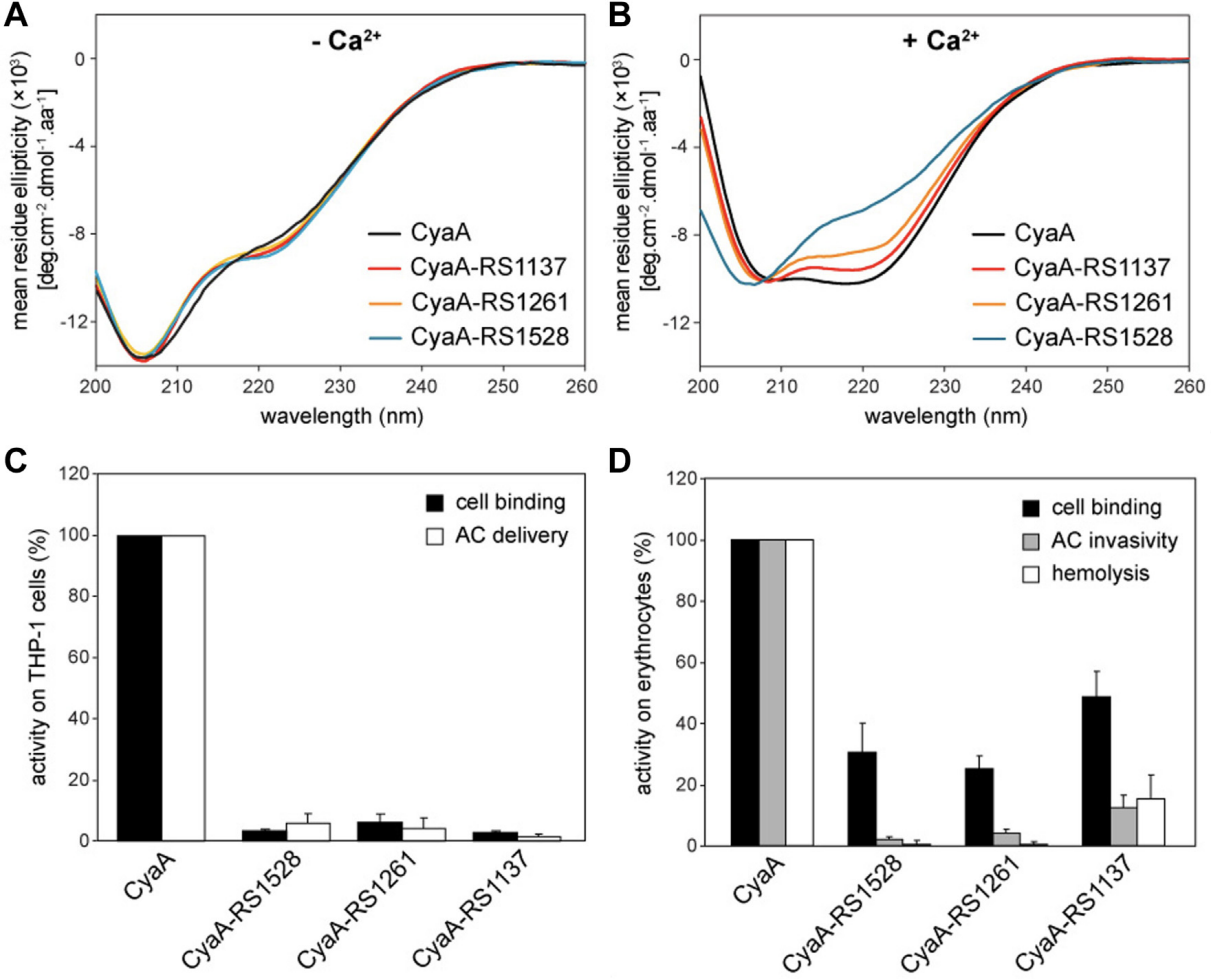
**Structural and functional properties of the RS-insertion CyaA mutants.***A* and *B*, far UV CD spectra of CyaA and its RS-insertion mutants in the absence of Ca^2+^(*A*) and in the presence of 2 mM Ca^2+^ ions (*B*). *C*, binding and cytotoxic activities of CyaA and its RS-insertion variants on THP-1 cells. The binding was determined in D-MEM (1.9 mM Ca^2+^) as the amount of cell-associated AC enzyme activity after incubation of 1 × 10^6^ cells with 1 μg/ml of the indicated CyaA proteins for 30 min at 4 °C. AC domain delivery of CyaA was assessed by determining the intracellular cAMP concentration generated at 37 °C in THP-1 cells (1.5 × 10^5^) after 30 min of incubation with four different CyaA concentrations from the linear range of the dose-response curve (250, 125, 62.5, and 31.25 ng/ml). The activities of CyaA at each of the toxin concentrations was taken as 100% for calculation of % of cAMP generated by CyaA-RS proteins used at each of the four indicated toxin concentrations. The % values were then averaged for each CyaA RS variant and the means are given. *D*, the activities of CyaA and its RS-insertion variants on red blood cells. Sheep erythrocytes (5 × 10^8^/ml) were incubated at 37 °C in the presence of 2 mM Ca^2+^ with 1 μg/ml of the purified CyaA proteins. After 30 min, aliquots were taken to determine the cell-associated AC activity (binding) and the AC activity internalized into erythrocytes and protected from digestion by externally added trypsin (Invasive AC). For determination of hemolytic activity, sheep erythrocytes (5 × 10^8^/ml) in TNC buffer were incubated at 37 °C with 10 μg/ml of CyaA proteins for 3 h as the amount of released hemoglobin was measured as A_541_. Activities are expressed as percentages of intact CyaA activity and represent averages ± SDs. N = 4. Two independent toxin preparations were analyzed in all assays. AC, adenylate cyclase; DMEM, Dulbecco’s modified Eagle’s medium.

To further examine the effect of folding-impaired RTX-iv on the overall structure of CyaA toxin, we incorporated the RS insertion into CyaA and obtained CyaA-RS1528. Its CD spectrum in the absence of Ca^2+^ shows the characteristics of an unfolded polypeptide chain (Fig. 6*A*), while in the presence of 2 mM Ca^2+^, CyaA-RS1528 acquired some folded β-roll structure but its fraction was significantly lower than in wt CyaA (Fig. 6*B*). This result is consistent with the expectation that RS1528 disrupted the folding of RTX-i-iv.

To examine if this RS-insertion approach can be extended to other RTX blocks, we also engineered RS insertion variants of RD and CyaA: RTX-i-v/CyaA-RS1137, in which RS was inserted between RTX-i and ii after residue 1137, and RTX-i-v/CyaA-RS1261, in which RS was inserted between RTX-ii and iii after residue 1261. Similar to the effect of RS1528, OT results (Fig. 5, *C* and *D*) showed that in RTX-i-v-RS1261, only the unfolding-refolding events of RTX-iii-v were observed while RTX-i-ii remained unfolded; and in RTX-i-v-RS1137, RTX-ii-v folded and only RTX-i remained unfolded. These results indicated that both RTX-i-v-RS1261 and RTX-i-v-RS1137 showed a similar disruptive effect on the folding of the RTX blocks located N-terminally to the RS insertion. Consistent with OT results, CD spectra of CyaA-RS1137 and CyaA-RS1261 showed that the fraction of the folded β-roll structures decreased from CyaA-RS1261 to CyaA-RS1137 in the presence of 2 mM Ca^2+^ (Fig. 6*B*).

Despite the clear changes of CD spectra and OT *F-D* curves of the RS variants, the thermal melting behaviors of these variants did not show significant changes compared with wt CyaA, as measured by nano differential scanning fluorimetry (nanoDSF) (Fig. S4). CyaA showed a broad thermal melting transition, suggesting that the thermal melting temperatures of individual RTX blocks are close to one another and are difficult to distinguish. This feature resulted in little change of the thermal melting curves of the RS variants. This result again highlights the challenges in using traditional calorimetry techniques to discern the unfolding and folding of the multiblock RD of CyaA and reveals the unique capability of OT in addressing this challenge.

Taken together, these results demonstrated that insertion of an RS dipeptide between two neighboring RTX blocks effectively disrupted the transmission of the folding signal within the RD and prevented the folding of the RTX blocks located N-terminally to the RS insertion. However, the detailed mechanism underlying this disruptive effect remains unknown. In order to decouple the RS effects on the β-strand folding from possible impact on the linker or changes to the block-block interface, systematic work, involving both experimental and simulation, will be needed to elucidate the key interactions in the linker sequence and the block-block interface that are important for this templating effect.

Since the CR3-binding interface of RD involves the linker regions between RTX-i and ii and between RTX-ii and iii, the disruption of the folding observed in the three RS insertion variants was expected to disrupt the formation of the CR3-binding site in RD and ablate toxin activities of CyaA. To test this prediction, we assayed the CyaA-RS variants for their capacity to bind CR3-expressing THP-1 human monocytic cells and deliver the ACD enzyme domain into cytosol of macrophages. Binding of the intact CyaA on THP-1 cells was dependent on the presence of CR3 and was strongly inhibited by the M1/70 mAb (Fig. S5) that recognizes the CyaA-binding site on CR3 (11, 24). Indeed, as shown in Figure 6*C*, all three CyaA-RS variants were devoid of these CyaA toxin activities on THP-1 cells. Hence, these results validate our prediction that the CyaA-RS variants did not have a well folded, functional CR3-binding site. In addition, abolishing the toxin activity by the RS1137 insert would suggest that both CR3-bindings regions (between RTX-i and ii as well as between RTX-ii and iii) (26) are involved in CyaA binding to CR3. Alternatively, insertion of the RS dipeptide at residue 1137 in the L1 linker of RD might have also disrupted the folding of the AR required for toxin function. Indeed, according to the recently solved cryo-EM structure, the AR contains two Ca^2+^-binding sites and folds as continuation of the β-roll structure of RTX-i (26), forming its contiguous N-terminal cap. Its folding would then assure the proper orientation of the C16 palmityl acid residues that are covalently linked to the ε-amino groups of lysine residues 860 and 983 in the L0 linker structure of RD (26). The irreversible insertion of the acyls of the modified K860 and K983 residues into the outer leaflet of the cell membrane appears to shift the association-dissociation equilibrium of the binding interaction of CyaA with its CR3 receptor to the right, stabilizing it compared to the nonacylated proCyaA binding (42). Hence, misfolding of the AR and misorientation of the attached acyls would likely impact on the measured toxin binding to the CR3 on THP-1 cells. The latter interpretation would go well with the observed defect of toxin activities of the CyaA-RS variants also on erythrocytes that do not possess the CR3 receptor molecule on cell surface (Fig. 6*D*). Indeed, the erythrocyte-binding activities of CyaA-RS1261 and CyaA-RS1528 were significantly reduced to ∼30% of wt CyaA but were only decreased by ∼50% for CyaA-RS1137 (Fig. 6*D*). It is important to note that the cell-invasive adenylate cyclase (AC) activity and hemolytic capacity of both CyaA-RS1528 and CyaA-RS1261 proteins were almost completely abolished, while CyaA-RS1137 still retained some level of both activities. This is striking, as CyaA can penetrate the membrane of erythrocytes and deliver the ACD enzyme in a CR3 receptor–independent manner (43). This result suggests that the RS insertions likely disrupted not only the folding of the RTX-blocks but also interfered with the functions of other regions of CyaA, most likely affecting the functional folding of the AR capping the β-roll structure of RTX-i (26). Hence, this result suggests that the folding of AR is templated by the folding of RTX-i.

It is also intriguing to note that CyaA-RS1137 with RS insertion in the L1 linker between blocks RTX-i and RTX-ii displays higher residual toxin activities and a higher fraction of folded RD than CyaA-RS1261 and -1528, indicating that folding of the L2 linker between blocks RTX-ii and RTX-iii generated enough of a CR3-binding surface to enable partial toxin binding and cell-penetrating activity on monocytes.

## Discussion

### The folding of the RD and AR is governed by the same templated folding mechanism

Our single molecule OT results unambiguously demonstrate that the folding of RD in the presence of Ca^2+^ is a hierarchical series process proceeding from the C- to N-terminus following a templated folding mechanism. The order in which RTX blocks fold follows a strict hierarchy, with the C-terminal RTX-v always being the first to fold. The folding of a given RTX block (RTX-ii to iv) is templated by its C-terminal neighboring RTX-block, and once folded, it also serves to template the folding of its N-terminal neighboring block. In addition, the biological activities of the RS-insertion variants suggest that the templating effect also extends to AR, that is, the folding of AR is templated by the folding of its C-terminal neighboring RTX-i. In other words, AR and RD can be considered as one large folding unit, and the folding signal is transmitted continuously from the C-terminus to the N-terminus *via* a cascade of templated folding events.

It is noteworthy that the folding of each individual RTX block in turn stabilizes its C-terminal neighboring RTX block, entailing a strict unfolding hierarchy among the RTX blocks. It is of particular note that the apparent mechanical stability of RTX-ii is considerably higher than that of other blocks. As the folding of RTX-ii completes the formation of the CR3-binding site between RTX-ii and RTX-iii, it is reasonable to speculate that the high mechanical stability of RTX-ii serves to stabilize the newly formed CR3-binding site.

Moreover, the folding of each individual RTX block occurred against a residual stretching force (a few pN). By analyzing the folding kinetics of individual RTX blocks using a well-established method (44), we determined the dependence of the folding/unfolding rate constant on the force, from which we extracted the folding/unfolding rate constant at zero force using the Bell-Evens model (45, 46) (Table S1). Our results showed that all RTX blocks can fold rapidly at zero force.

The sum of these experimental evidence indicates that *in vitro*, the folding of RD occurs rapidly *via* a templated folding mechanism, resulting in vectorial folding of RD in the direction from C- to N-terminus, which coincides with the direction of secretion starting with C-terminal segment. Such a vectorial folding is an intrinsic property of RD and is driven by the binding of extracellular Ca^2+^ ions upon exiting from the T1SS conduit and does not require any other cofactors.

These insights as well as the unique features of the secretion process of CyaA under physiological conditions suggest that the folding of RD under its physiological condition follows a cosecretional vectorial folding mechanism. Along the secretion direction (from C- to N-termini), the secreted RTX polypeptide can bind Ca^2+^ and rapidly fold into the β-roll structure as soon as it emerged from the T1SS conduit. The mechanical stability of the refolded RTX block helps itself remain folded and prevents its backsliding. The folded RTX block can then serve a template to facilitate the folding of its N-terminal block that newly emerged from the T1SS conduit.

Since the folding direction (from C- to N-terminus) of the RD and AR is the same as the direction of their secretion to the extracellular space, these results validate that under physiological conditions in host body fluids containing 2 mM Ca^2+^, the folding of RD follows a Ca^2+^-driven, cosecretional vectorial folding mechanism, as proposed earlier by Bumba *et al.* (16) and later modified by Wang *et al.* (17). This mechanism can now be extended to both AR and RD. Along the secretion direction (from C- to N-termini), once the secreted RTX polypeptide chain emerges from the T1SS conduit on bacterial cells surface, the binding of Ca^2+^ and the template effect will enable individual RTX blocks in RD and AR to fold sequentially and rapidly in a vectorial manner (Table S1). The mechanical stability of the folded RTX blocks helps themselves to remain folded and prevents backsliding of the extruded CyaA polypeptide in the T1SS conduit. Each folded RTX block can then serve as a template to facilitate the folding of the subsequently emerging N-terminally located RTX block exiting from the T1SS conduit. In addition, the ability of RTX blocks to fold against a few pN residual forces would allow the folding of a given β-roll structure to generate a pulling force, facilitating translocation of the CyaA polypeptide chain in the T1SS conduit and effectively accelerating the extrusion process. When combined, these individual effects collectively make the secretion process highly efficient.

### Continuous templated folding has important biological implications

The templated folding mechanism in RD and AR indicates that the folding signal in the AR and RD region of CyaA is transmitted *via* a relay pathway from the C-terminal towards the N-terminal end of the protein. Although this unique pathway helps CyaA to achieve high efficacy of secretion/folding and consequently helps CyaA to acquire its toxin activity, the series nature of this transmission pathway also makes CyaA vulnerable to potential disruption. The failure of folding any preceding β-roll block in the AR and RD region can interrupt this folding signal transmission pathway, leading to the disruption of the folded structure of the N-terminal end of the protein and loss of biological function of CyaA. This intrinsic vulnerability may open up new opportunities for design/screening of new small molecule inhibitors of RD folding that could abolish CyaA toxin activity, as disrupting the folding signal at any given RTX block could cut off the folding transmitting pathway and significantly disrupt CyaA both structurally and functionally. On the one hand, a disruption (either the RS insertion or the deletion of the C-cap) can cut off the transmitting of the folding signal and thus cause the sequence of AR and RD, which is at the N-terminal upstream of the disruption site, to lose its ability to fold and remain unstructured after secreted into the extracellular milieu. On the other hand, the structural disruption results in the disruption/abolishment of CyaA’s toxin activity. The disruption of the CR3-binding site abolishes the cell-binding capability of CyaA, and an unfolded AR significantly reduces the ability of CyaA to insert into host membrane. Both effects synergistically help abolish the capacity of CyaA to deliver ACD into host macrophagocytes, leading to the significant reduction or even a complete abolishment of CyaA’s toxin activities.

Although the series templated folding mechanism we report here is demonstrated for CyaA, it is tempting to speculate that this mechanism is general for other large members of the RTX family that possess several RTX blocks separated by comparable linker sequences like CyaA. Previous studies showed that the essential role played by the C-terminal RTX capping structure for the folding and biological activities of CyaA is a general feature for other RTX leukotoxin family proteins (16). Since the folding of the C-terminal capping structure can be considered as the very first step of the series of templated folding events, it is likely that this templated folding mechanism is a general feature shared by other large members of the RTX family or at least close relatives of CyaA. However, future experiments are needed to validate this prediction.

Our results now unravel the possibility to disrupt the transmission pathway of the folding signal in the hemolytic region of CyaA at multiple sites along the sequence of the RD. The fast development of computational biology tools thus opens the way to targeting of templating interfaces between any two neighboring RTX blocks, such as the one between RTX-iv and v as well as RTX-iii and iv. This should allow the design and screening for inhibitors capable of binding RD and disrupting a specific templating interface to block the folding of its N-terminal sequence and abolish the toxin activity of CyaA.

## Experimental procedures

### Protein engineering

The gene encoding the whole RD (pET42b/RTX-i-v) was constructed as reported (16). The genes encoding RTX-iii-v and RTX ii-v were amplified from pET42b/RTX-i-v using PCR technique. The restriction sites (5′ *Bam*HI and 3′ *Kpn*I) were introduced to the genes *via* the designed primer for PCR reaction. The amplified genes were then ligated to a custom-engineered vector, pETcc, which contains a cassette encoding a 5′ Cys-NuG2 and 3′ NuG2-Cys, *via* the BamHI and KpnI restriction sites. The obtained genes are as follows: pETcc/Cys-NuG2-RTX-iii-v-NuG2-Cys, pETcc/Cys-NuG2-RTX-ii-v-NuG2-Cys, and pETcc/Cys-NuG2-RTX-i-v-NuG2-Cys, respectively.

The mega primer approach was used to engineer the RS insertion RD variants using appropriate RTX construct (RTX-i-v, RTX-ii-v and RTX-iii-v and RTX-iv-v) as the template for amplification. Residue number 1137, 1261, and 1528 was used according to the location in the CyaA sequence.

The expression vectors encoding the CyaA-RS1528, CyaA-RS1261, and CyaA-RS1137 proteins were derived from pCACT3, a bicistronic vector encoding the structural *cyaA* gene and the *cyaC* gene for the dedicated toxin acyltransferase (47). For assembly of the pCACT3-RS1528 vector, two PCR products amplified from the pCACT3 plasmid using a pair of primers (5′-CAAGTGGTGGAGGTCGACACGCTCGAGCATGTGCAGCAC-3′, 5′-ACGTCATCA CGCGCGCTGCCGCTACGCACGGCGTTCTCGATATTGC-3′, and 5′-GCAATATCGAGAACGC CGTGCGTAGCGGCAGCGCGCGTGATGACGT-3′, 5′-ATGCTTTTCTGTGACTGGTGAGTACT CAACCAAGTCATTCTG-3′) were inserted into the ScaI/XhoI-cleaved fragment of the pCACT3 (5637 bps).

The pCACT3-RS1261 vector was prepared by ligation of the XhoI/XmaI-cleaved fragment of pCACT3 (7442 bps) with the XhoI/XmaI-digested PCR product amplified using a pair of primers (5′-ACGCTCGAGCATGTGCAG-3′ and 5′-CGGCCCGGGCCGCTG-3′) from two PCR products as templates, each obtained by the PCR amplification of the DNA sequence upstream (5′-ACGCTCGAGCATGTGCAG-3′ and 5′-CATCCTTCATGCTCGTACCGGAGCGGATGACGTTTT CGACATTGC-3′) and downstream (5′-GCAATGTCGAAAACGTCATCCGCTCCGGTACGAG CATGAAGGATG-3′ and 5′-CGGCCCGG GCCGCTG-3′) of the insertion site.

The pCACT3-RS1137 vector was prepared by ligation of the XhoI/XmaI-cleaved fragment with the XhoI/XmaI-digested PCR product amplified using a pair of primers (5′-ACGCTCGAGCATGTGCAG-3′ and 5′-CGGCCCGGGCCGCTG-3′) from two PCR products as templates, each obtained by the PCR amplification of the DNA sequence upstream (5′-ACGCTCGAGCATGTGCAG-3′ and 5′-GTCGTTTAGGCGGGAGCCGGAGCGGTGCAGATTCT CGATATTCTT-3′) and downstream (5′-AAGAATATCGAGAATCTGCACCGCTCCGGCTCCCG CCTAAACGAC-3′ and 5′-CGGCCCGGGCCGCTG-3′) of the insertion site.

### Protein purification and characterization

The recombinant proteins were overexpressed in *Escherichia coli* strain BL21(DE3) at 37 °C in 200 ml 2.5% LB media with 100 mg/l ampicillin. One millimolar of IPTG (Thermo Fisher Scientific) was added to induce the overexpression when the optical density of the culture reaches 0.7 ∼ 0.8. The overexpression continued for 4 h at 37 °C, 225 r.p.m. The bacteria cell pellets were then separated by centrifugation at 5000 r.p.m. (4360*g*) at 4 °C for 10 min. The protein was then extracted using lysozyme lysis method and was purified *via* Co^2+^ affinity. The protein was eluted and stored in elution buffer (10 mM PBS, 300 mM NaCl, 250 mM imidazole). The purified apo-form RTX polyprotein was at a concentration of ∼1.0 mg/ml and stored at −80 °C.

Full-length CyaA proteins were produced in *E. coli* XL-1 Blue (Stratagene) in MDO medium (yeast extract, 20 g/l; glycerol, 20 g/l; KH_2_PO_4_, 1 g/l; K_2_HPO_4_, 3 g/l; NH_4_Cl, 2 g/l; Na_2_SO_4_, 0.5 g/l; and thiamine hydrochloride, 0.01 g/l) supplemented with 150 μg/ml of ampicillin, induced at A_600_ = 0.6 with 1 mM IPTG, and grown for additional 4 h. CyaA proteins were purified from urea extract by single-step chromatography on DEAE Sepharose.

### Single-molecule optical tweezer experiment

The preparation of dsDNA and DNA-protein chimera were carried out using mini-tweezers setup following well-established protocol as previously described (29, 48). One microliter of streptavidin-coated polystyrene beads (1% w/v 1 μm, Spherotech Inc) was diluted by 3 ml Tris–HCl buffer (20 mM Tris, 150 mM NaCl, pH 7.4) and injected into a fluid chamber. One single streptavidin-coated bead was trapped by the laser beam and moved onto the tip of a micropipette. The bead was then held by the micropipette by applying vacuum. Four microliters of anti-digoxigenin–coated polystyrene beads (0.5% w/v, 2 μm, Spherotech Inc) was mixed with 1 μl 10 nM DNA-protein chimera for 30 min. The anti-dig beads coated with DNA-protein chimera was then diluted to 3 ml and injected to the chamber. The sample-coated anti-dig bead was captured by the optical trap and brought into contact with the streptavidin bead held by the micropipette to establish the bead-DNA–protein dumbbell *via* the biotin–streptavidin interaction. By moving the laser trap, the target protein could be stretched (or relaxed) to induce the mechanical unfolding (or folding) (Fig. 6). For experiments on apo-RTX, Tris–HCl buffer (20 mM Tris, 150 mM NaCl, pH 7.4) was used. For holo-RTX protein, the same Tris–HCl but supplemented with 10 mM Ca^2+^ was used.

### Cell lines

Human monocytes THP-1 (ATTC number TIB-202) were cultured in RPMI medium supplemented with 10% heat-inactivated fetal bovine serum and antibiotic/antimycotic solution (Sigma). Prior to assays, RPMI medium was replaced with Dulbecco’s modified Eagle’s medium (DMEM, 1.9 mM Ca^2+^) without fetal bovine serum.

### Determination of AC enzyme activity

Enzymatic AC activity was measured in the presence of calmodulin (1 μM) as described previously (49). One unit of AC corresponds to 1 μmol cAMP formed in 1 min at pH 8 at 30 °C.

### Binding and cell-invasive activity of CyaA on sheep RBCs

Sheep RBCs (LabMediaServis) were washed in TNC buffer (50 mM Tris–HCl at pH 7.4, 150 mM NaCl, 2 mM CaCl_2_), adjusted to 5 × 10^8^ cells/ml, and incubated with CyaA for 30 min at 37 °C in TNC buffer. One aliquot of cells was used after extensive washing in cold 50 mM Tris–HCl (pH 7.4), 150 mM NaCl, and 5 mM EDTA (TNE buffer) to determine the amount of cell-associated AC activity (binding). The second aliquot was incubated at 37 °C with trypsin (20 μg/ml, 15 min) to inactivate the extracellular AC enzyme that was not translocated to the cell cytosol. Soybean trypsin inhibitor (40 μg/ml) was then added to the mixture to stop the reaction before samples were washed twice in cold TNE buffer and used to determine the AC enzyme translocated into cells (invasive AC).

### Hemolytic activity

Hemolytic activity was measured at 37 °C in TNC buffer as hemoglobin release (A_541_) from washed sheep erythrocytes (5 × 10^8^ cells/ml).

### Binding of CyaA to nucleated cells

THP-1 cells (10^6^/ml) were incubated in D-MEM with CyaA (1 μg/ml) for 30 min at 4 °C. Unbound toxin was removed by washing three times with DMEM, cells were lysed with 0.1% Triton X-100, and membrane-associated AC enzyme activity was determined by AC enzyme activity assay as described previously (49).

### cAMP determination

THP-1 cells (1.5 × 10^5^/well in D-MEM) were incubated for 30 min at 37 °C with various concentrations of CyaA proteins from the linear range of the dose-response curve and the enzymatic reaction was stopped with 0.2% Tween-20 in 100 mM HCl. Samples were boiled at 100 °C for 15 min, neutralized with 150 mM unbuffered imidazole, and cAMP was measured by competitive immunoassay as described previously (50, 51).

### CD spectroscopy

The far-UV CD spectra were recorded at 25 °C on a Chirascan-plus spectrometer (Applied Photophysics) in rectangular quartz Suprasil cells of 1-mm path length (110-QS, Hellma). The Ca^2+^-free protein samples (0.1 mg/ml) were diluted in 20 mM Tris–HCl (pH 8.0) and 50 mM NaCl in the absence or the presence of 2 mM CaCl_2_ and measured for wavelengths from 200 to 260 nm with a scanning speed of 1 nm/s. Spectra of the buffers were subtracted from the protein spectra, and the mean residue ellipticity (Θ) was expressed in degrees square centimeter per decimole and amino acid residue [deg.cm^2^.dmol^−1^.aa^−1^].

### NanoDSF

Thermal stability assays were performed by a nanoDSF using a Prometheus NT.48 device (NanoTemper Technologies). The proteins were diluted in a final concentration of 0.1 mg/ml in buffer containing 50 mM Tris–HCl (pH 8.0), 150 mM NaCl, and 2 mM CaCl_2_ and loaded into nanoDSF grade standard capillaries (NanoTemper Technologies). The measurements were conducted from 20 to 95 °C (with a temperature ramp of 1.5 °C/min) under constant monitoring of tryptophan fluorescence at 350 and 330 nm. Data were processed and analyzed by using a PR.ThermControl software (NanoTemper Technologies, https://nanotempertech.com/prometheus-pr-thermcontrol-software/).

## Data availability

All data pertinent to this work are contained within this manuscript or available upon request. For requests, please contact: Hongbin Li, hongbin@chem.ubc.ca.

## Supporting information

This article contains supporting information.

## Conflict of interest

The authors declare that they have no conflict of interests with the contents of this article.
