# Supplementary material for: The adenylate cyclase toxin RTX domain follows a series templated folding mechanism with implications for toxin activity
Source: J Biol Chem. 2023 Aug 9;299(9):105150. doi: 10.1016/j.jbc.2023.105150 (PMC10511787; doi:10.1016/j.jbc.2023.105150)
Supplement: Supporting information [file mmc1.pdf]

Supporting Information

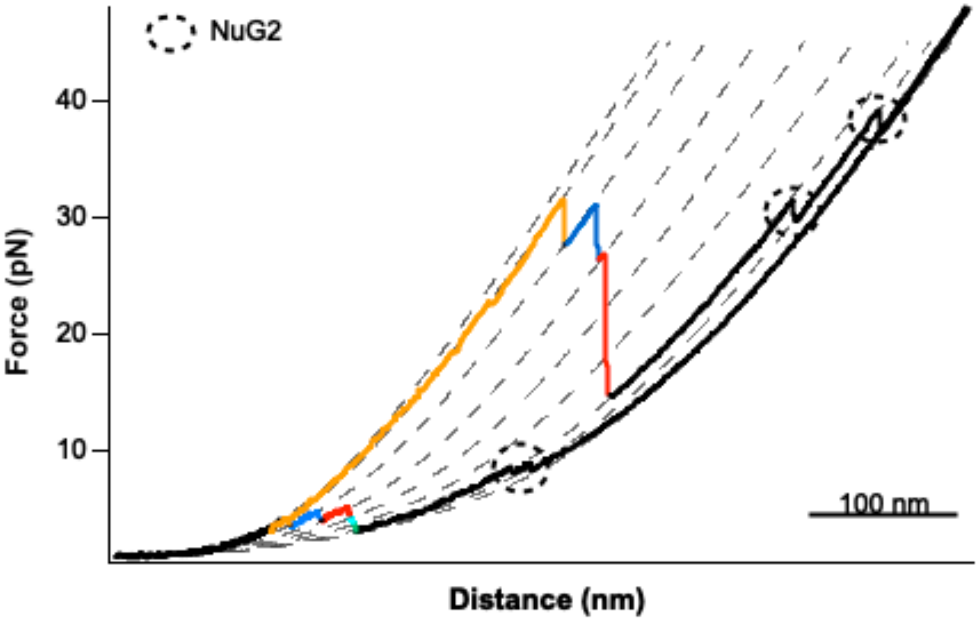

Figure S1. Force-Distance curves of full length NuG2-RTX-i-v-NuG2. The unfolding and refolding of the fingerprint domain NuG2 (circled) show a characteristic ΔLc of ~18 nm. The refolding of NuG2 domain occurs at ~ 7 pN, while the unfolding often occur after all RTX blocks have unfolded.

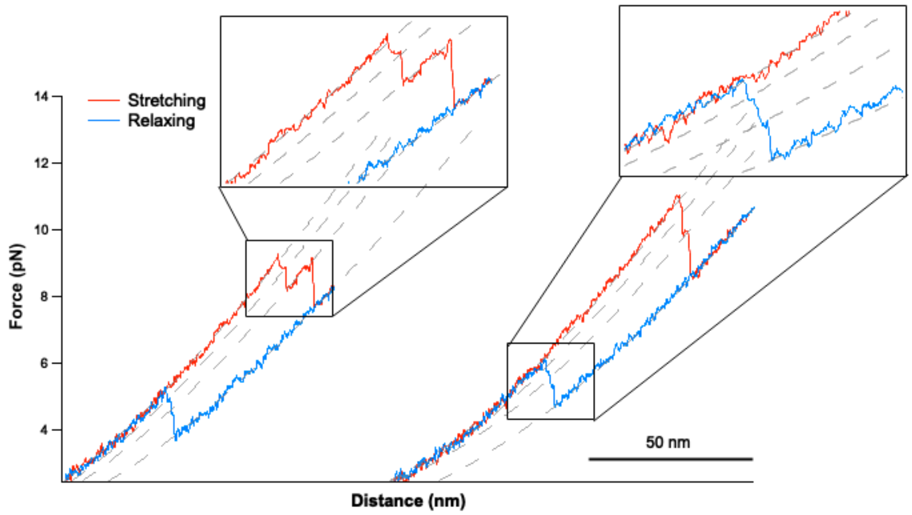

Figure S2. Unfolding and refolding of RTX-iv involve multiple intermediate states.

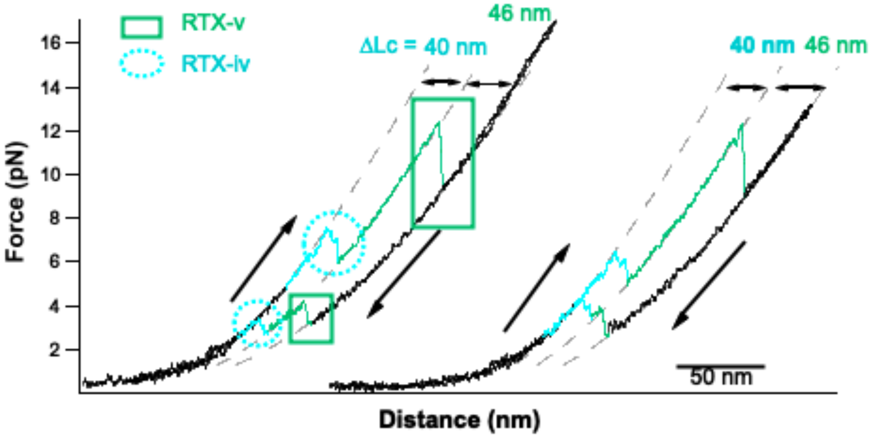

Figure S3. Force-distance curves of RTX-iv-v show the hierarchical order of unfolding and refolding of RTX-iv and v. During mechanical unfolding, RTX-iv unfolds first followed by RTX-v, but during refolding, RTX-v refolds first followed by RTX-iv. For convenience, the unfolding/folding events of RTX-iv and v are colored in cyan and green, respectively.

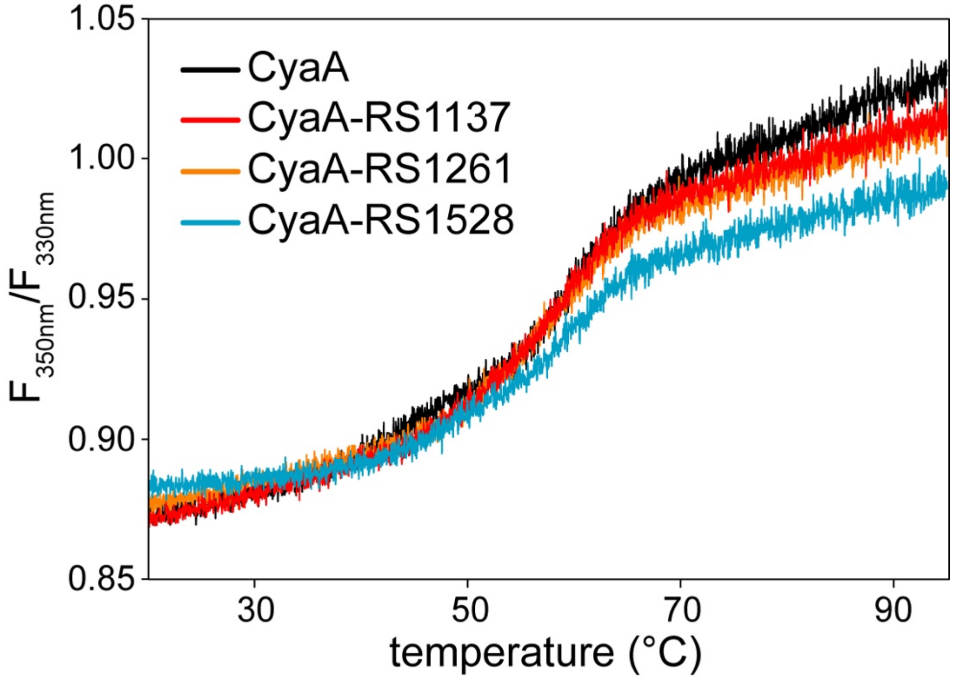

Figure S4. NanoDSF thermal melting curves of wt CyaA and its RS-insertion variants CyaA-RS1137, RS1261 and RS1528.

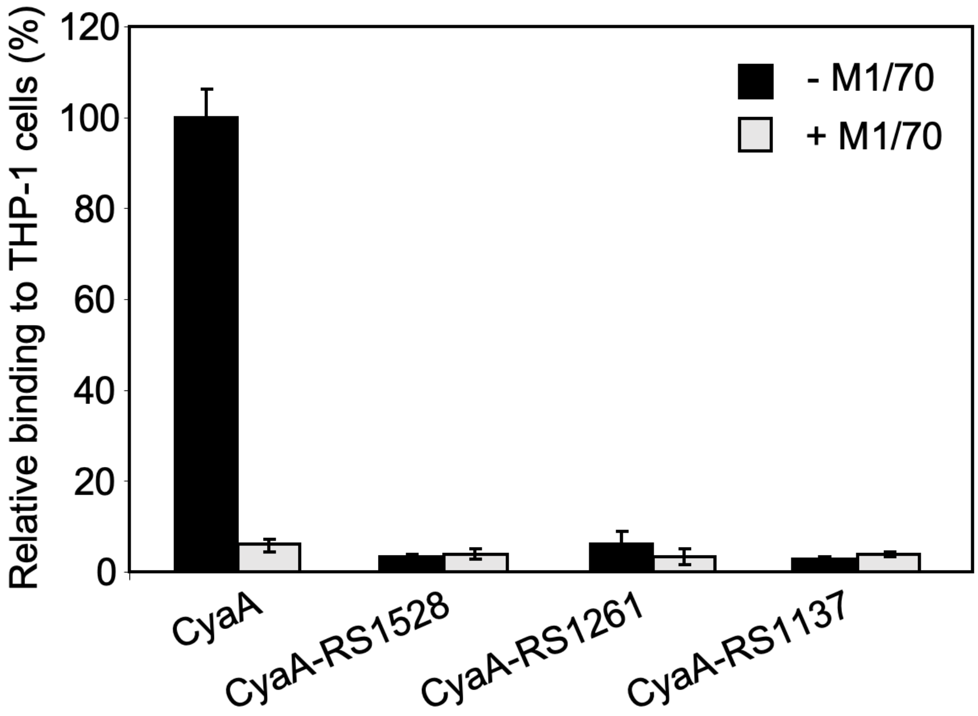

Figure S5. The binding of intact CyaA or its mutant variants to THP-1 cells ($1x10^6$) was determined in D-MEM medium as the amount of total cell-associated AC enzyme activity after incubation of cells with 1 µg/ml of the protein for 30 min at 4°C. To block the CR3, THP-1 cells were incubated on ice with 5 µg/ml of the CD11b-specific monoclonal antibody M1/70 (Pharmingen) for 15 min prior to addition of the CyaA variants. Activities are expressed as percentage of the intact CyaA activity and represent averages ± standard deviations. Two independent toxin preparations were analyzed. N=3.

Table S1. Kinetic parameter of RTX-i, RTX-ii, and RTX-iii

		$\alpha_0\ (s^{-1})$	$\Delta x_u\ (nm)$	$\beta_0\ (s^{-1})$	$\Delta x_f\ (nm)$
RTX-i	N to I	$(1.3 \pm 0.9) \times 10^{-1}$	1.5 ± 0.3	32.1 ± 6.4	4.5 ± 0.2
	I to U	$(3.5 \pm 0.6) \times 10^{-2}$	0.6 ± 0.03	$(1.4 \pm 1.2) \times 10^{3}$	9.3 ± 0.9
RTX-ii		$(1.6 \pm 0.4) \times 10^{-3}$	1.0 ± 0.04	$(1.4 \pm 1.0) \times 10^{3}$	7.8 ± 0.6
RTX-iii		$(5.7 \pm 4.1) \times 10^{-5}$	3.1 ± 0.2	$(1.5 \pm 1.2) \times 10^{2}$	4.8 ± 0.9

α_0: unfolding rate constant at zero force
β_0: folding rate constant at zero force
Δx_u: the distance between the native state and the mechanical unfolding transition state
Δx_f: the distance between the unfolded state and the folding transition state
Kinetic parameters were obtained by fitting the force-dependency of the logarithmic of (un)folding rate constant using the Bell-Evens model.
